# Supplementary material for: Affinity proteomics within rare diseases: a BIO-NMD study for blood biomarkers of muscular dystrophies
Source: EMBO Mol Med. 2014 Jun 11;6(7):918–36. doi: 10.15252/emmm.201303724 (PMC4119355; doi:10.15252/emmm.201303724)
Supplement: Supplementary file 1 — Supplementary Figure S1 [file emmm0006-0918-SD1.pdf]

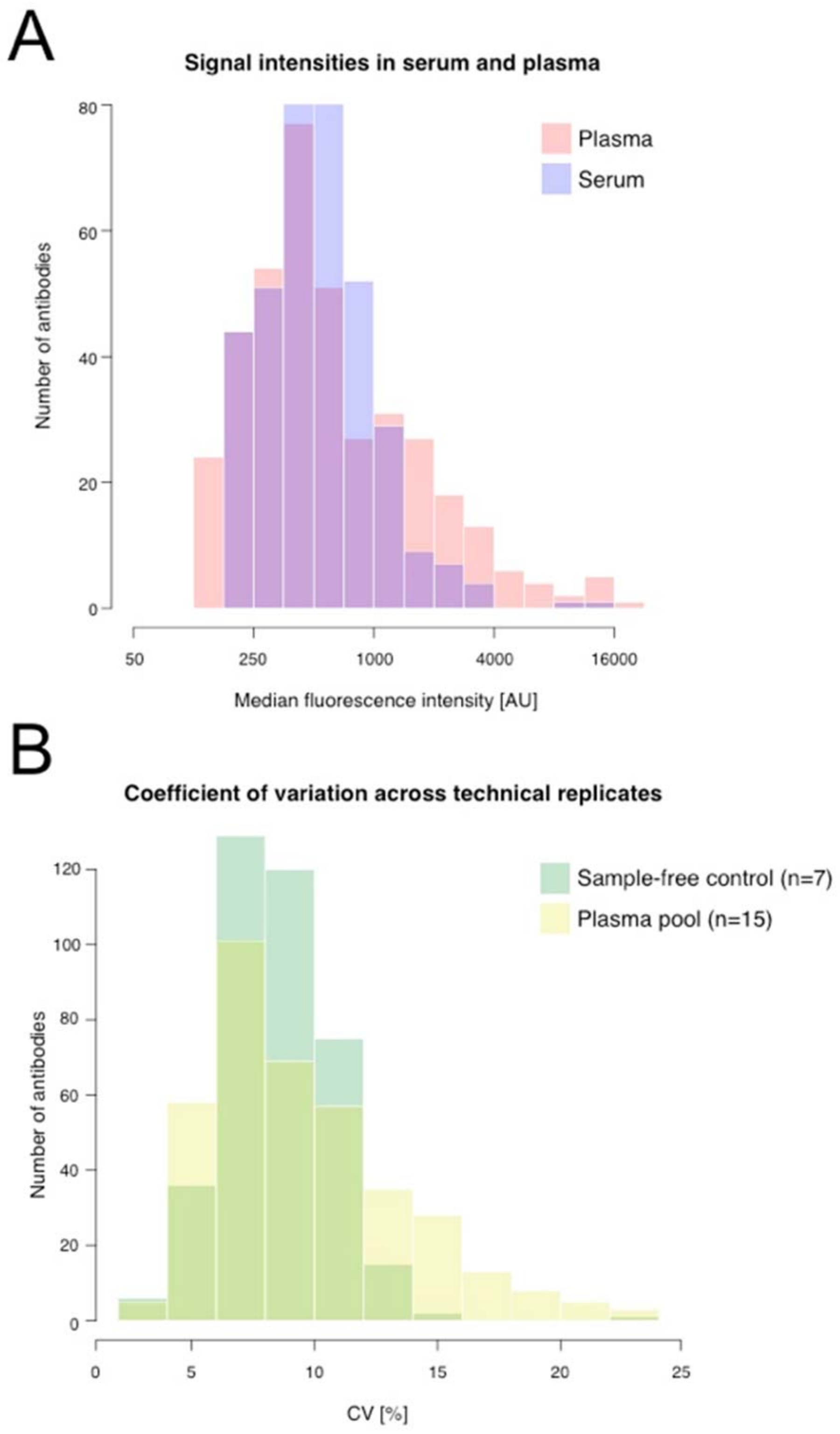

Supplementary Figure S1. Distribution of average signal intensity levels in serum/plasma and intra-assay variance. A histogram of average MFI values over 225 plasma and 120 serum samples for each of the 384 antibodies (**A**) and histogram of percentage of coefficient of variation (CV) in two different types of technical replicates **(B)**. The technical quality of the assay was assessed by the intra-assay CV using MFI values over 7 replicates of a sample-free control and 15 replicates of pooled plasma samples. The median

CV of all the antibodies across replicates of the sample-free control and of a pooled plasma sample were 8% and 9%, respectively. For the plasma pool, there were only 10 antibodies with a CV > 20%, whereas there was a single antibody with a CV > 20% in the sample-free control.
