# Supplementary material for: Affinity proteomics within rare diseases: a BIO-NMD study for blood biomarkers of muscular dystrophies
Source: EMBO Mol Med. 2014 Jun 11;6(7):918–36. doi: 10.15252/emmm.201303724 (PMC4119355; doi:10.15252/emmm.201303724)
Supplement: Supplementary file 2 — Supplementary Figure S2 [file emmm0006-0918-SD2.pdf]

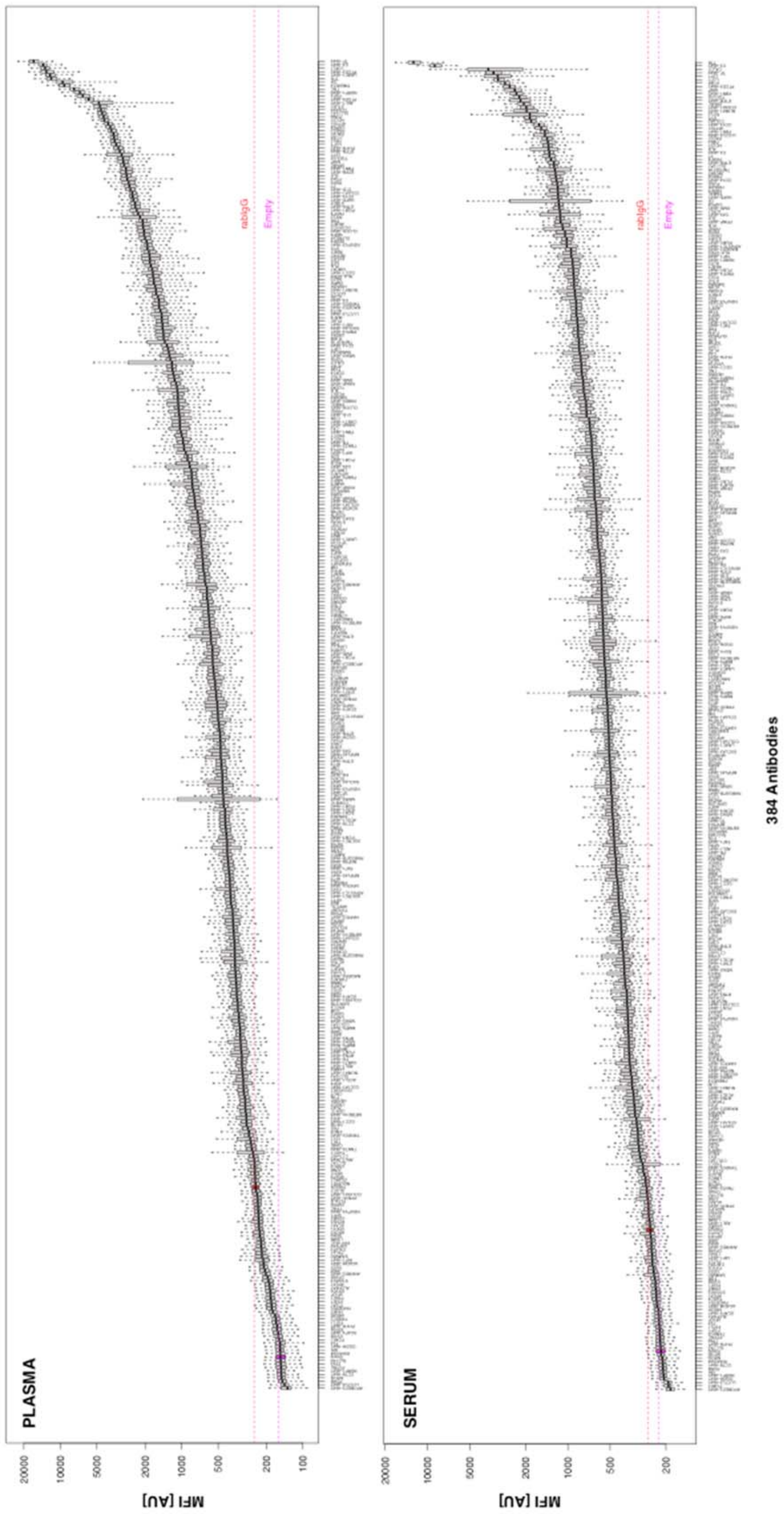

Supplementary Figure S2. Overview of signal intensity levels per antibody in serum/plasma. Boxplots represent the MFI values over all serum or all plasma samples. The upper endpoints of the 99% confidence intervals for the negative control beads coupled either

to no antibody ("Empty") or to non-specific rabbit IgG ("rabIgG") are shown with violet and red dashed lines, respectively. 96% and 94% of the antibodies displayed signal intensities over the empty bead and 83% and 86% of them displayed signal intensities over the rabbit IgG bead in plasma and serum, respectively. For each antibody, the box-and-whisker plot represents signal intensities within lower and upper quantile (box), the median (horizontal line within box), percentiles of 5% and 95% (whiskers) and outliers (dots).
