# Supplementary material for: Affinity proteomics within rare diseases: a BIO-NMD study for blood biomarkers of muscular dystrophies
Source: EMBO Mol Med. 2014 Jun 11;6(7):918–36. doi: 10.15252/emmm.201303724 (PMC4119355; doi:10.15252/emmm.201303724)
Supplement: Supplementary file 3 — Supplementary Figure S3 [file emmm0006-0918-SD3.pdf]

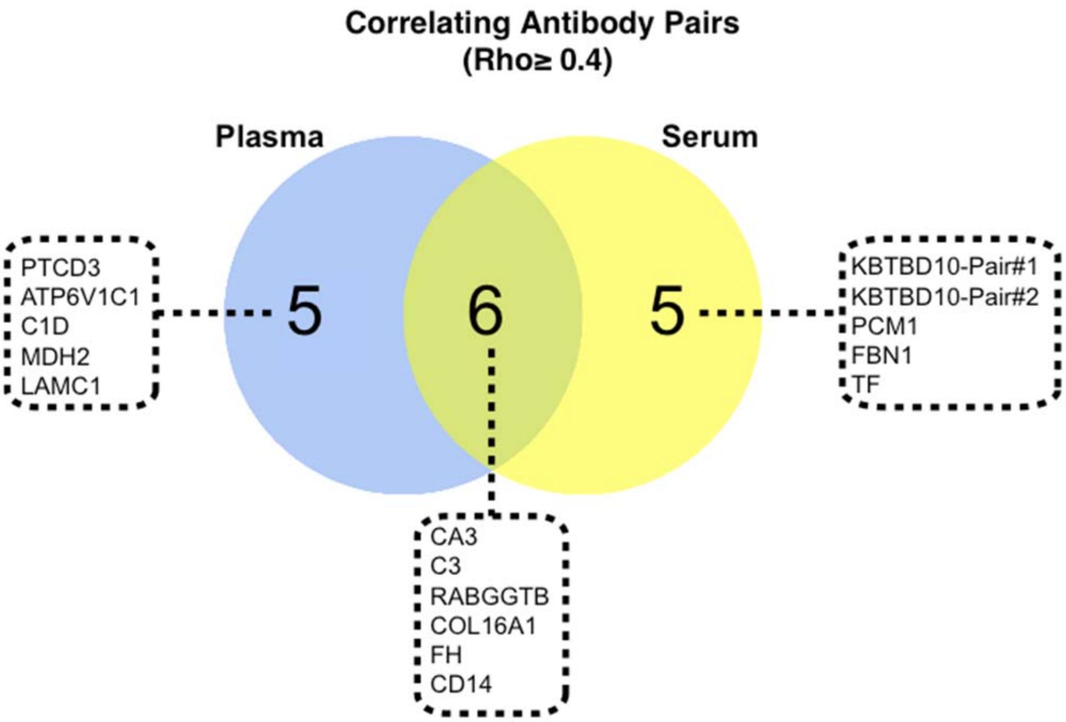


**Supplementary Figure S3. Number of correlating antibody pairs targeting different parts of same protein in serum and plasma.** As detailed in *Supplementary Table S1*, the antibody set included two or more antibodies produced against different parts of 56 of the 315 target proteins, resulting in 75 such antibody pairs. Despite the fact that not all the targets are necessarily detectable and that the majority of the antibody pairs target different epitopes/isoforms with different accessibility, 16 revealed a Spearman's Rho ≥ 0.4 either in serum or plasma, with 6 of these pairs being common for both blood preparation types. The 6 targets for which we observed well-correlating protein profiles both in serum and plasma included one of the highlighted candidates, namely CA3 (Spearman's Rho in serum=0.83, in plasma=0.80), whereas the antibody pairs targeting MDH2 revealed correlating protein profiles only in plasma (Spearman's Rho=0.44) but not in serum.
