# Supplementary material for: Affinity proteomics within rare diseases: a BIO-NMD study for blood biomarkers of muscular dystrophies
Source: EMBO Mol Med. 2014 Jun 11;6(7):918–36. doi: 10.15252/emmm.201303724 (PMC4119355; doi:10.15252/emmm.201303724)
Supplement: Supplementary file 4 — Supplementary Figure S4 [file emmm0006-0918-SD4.pdf]

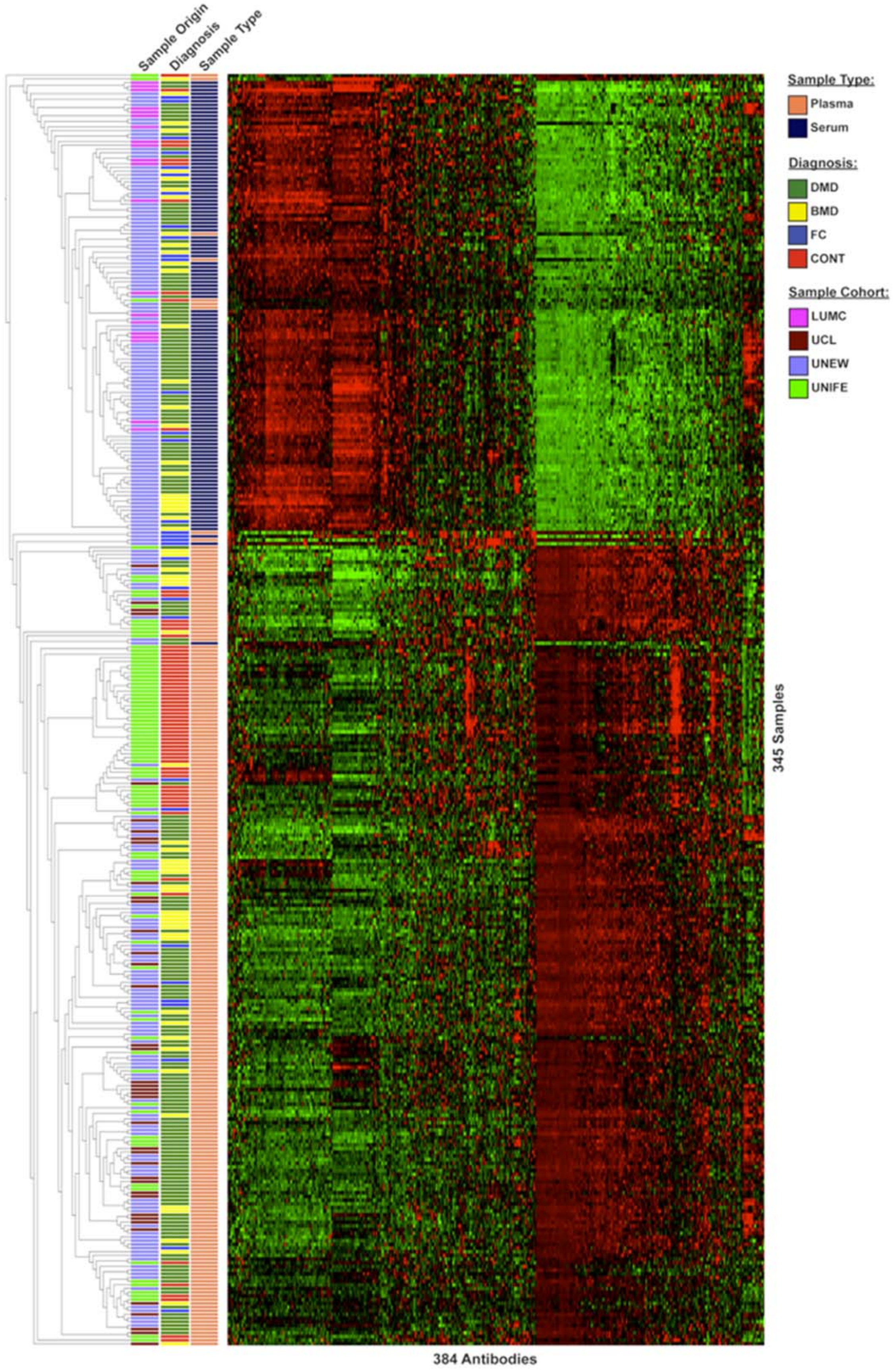

Supplementary Figure S4. Effect of blood preparation type and sample origin on protein profiles. Hierarchical clustering of all 345 samples and 384 antibodies included in the study, where samples are color-annotated according to blood preparation type, diagnosis and sample origin in the dendrogram.
