# Supplementary material for: Affinity proteomics within rare diseases: a BIO-NMD study for blood biomarkers of muscular dystrophies
Source: EMBO Mol Med. 2014 Jun 11;6(7):918–36. doi: 10.15252/emmm.201303724 (PMC4119355; doi:10.15252/emmm.201303724)
Supplement: Supplementary file 5 — Supplementary Figure S5 [file emmm0006-0918-SD5.pdf]

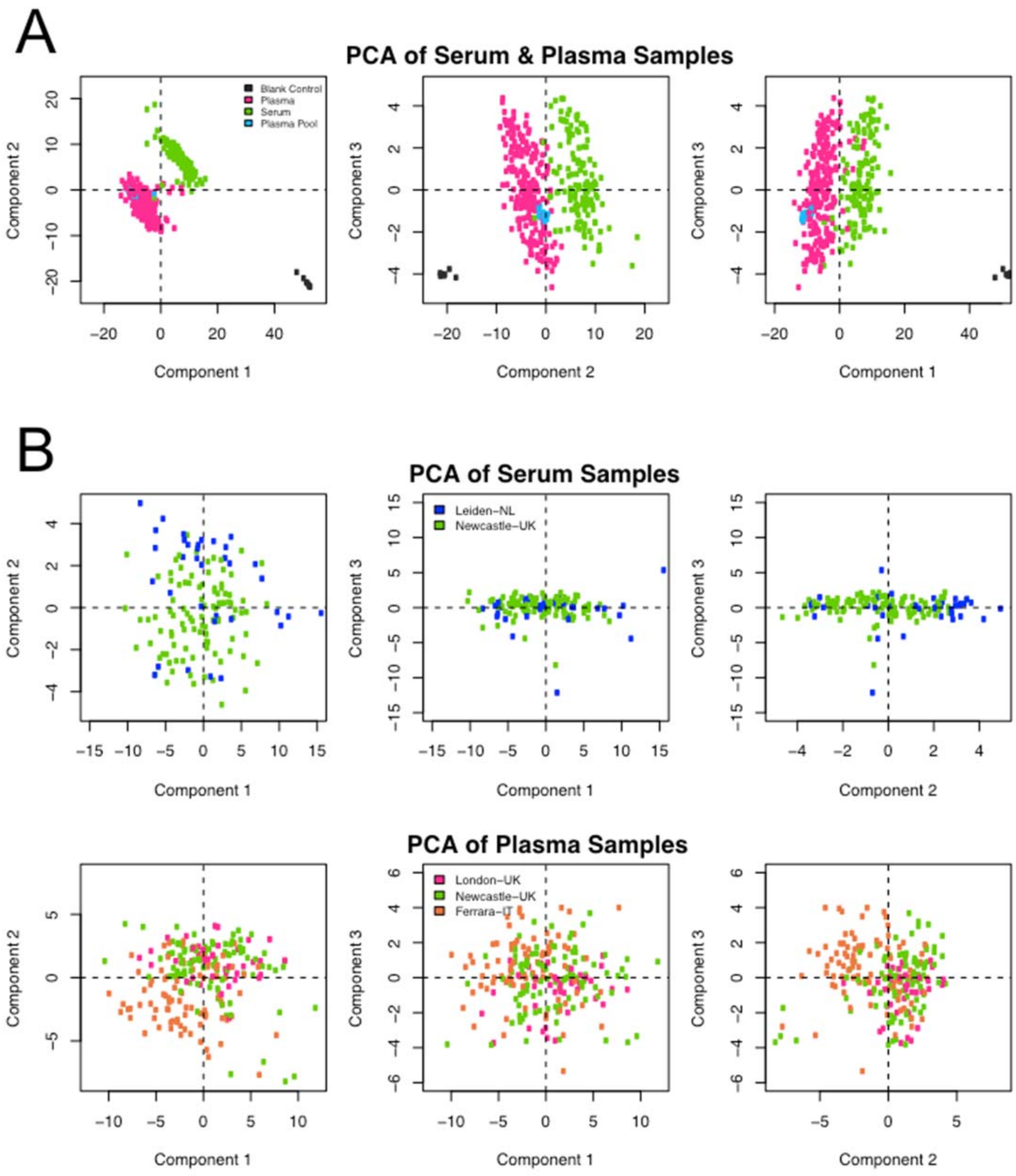

Supplementary Figure S5. Principal component analysis (PCA) of the entire sample set including all plasma and serum samples and assay controls and PCA of only serum or only plasma samples collected at different clinical sites. PCA revealed that the protein profiles across all plasma and serum samples collected at different sites grouped mainly by the blood preparation type (**A**). Furthermore, despite the standardized sample collection, handling and storage protocols adopted within the BIO-NMD consortium, a slight effect of sample origin was observed where the plasma samples originating from Ferrara, Italy varied slightly from the ones collected in London or Newcastle, UK (**B**). Such a difference might be explained due to exposure of the specimen to temperature fluctuations during transit.
