# Supplementary material for: Affinity proteomics within rare diseases: a BIO-NMD study for blood biomarkers of muscular dystrophies
Source: EMBO Mol Med. 2014 Jun 11;6(7):918–36. doi: 10.15252/emmm.201303724 (PMC4119355; doi:10.15252/emmm.201303724)
Supplement: Supplementary file 6 — Supplementary Figure S6 [file emmm0006-0918-SD6.pdf]

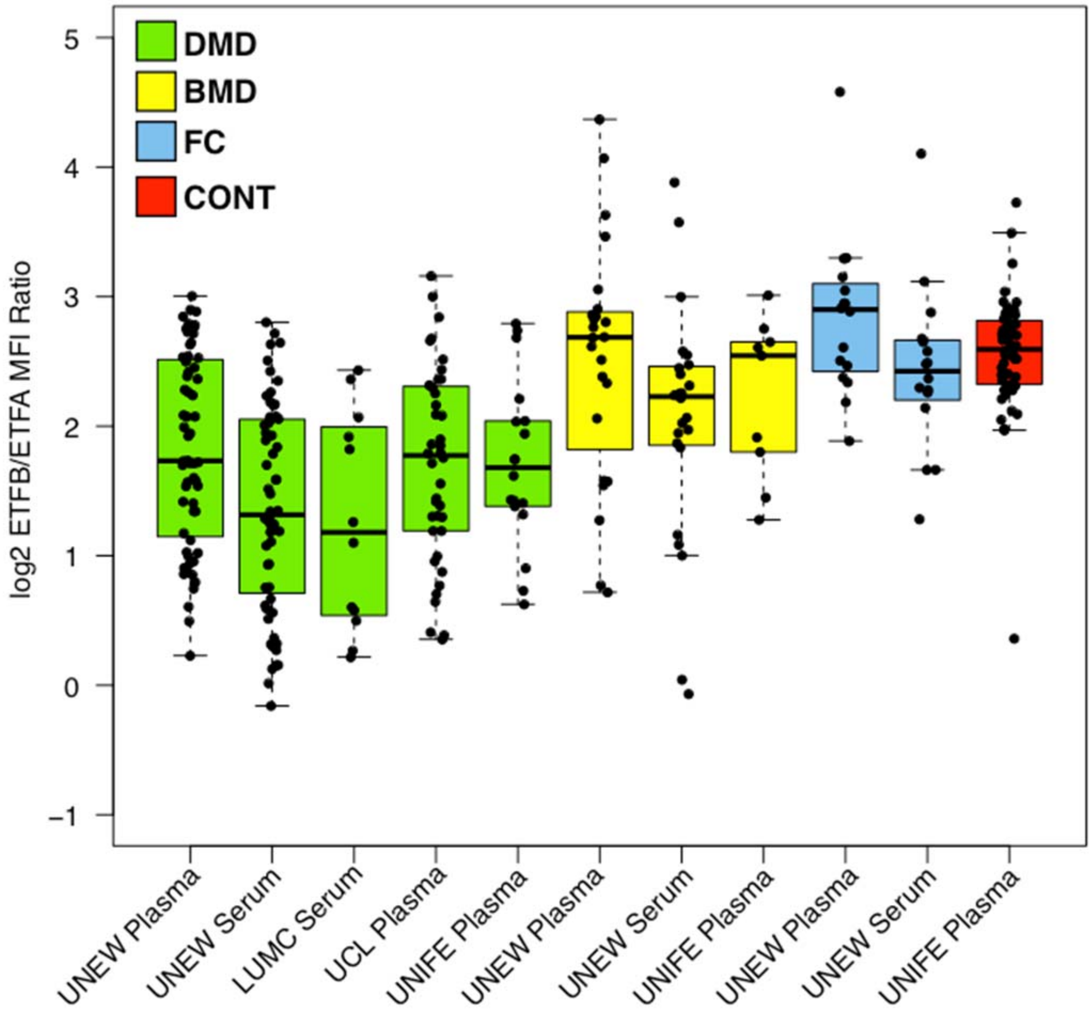

Supplementary Figure S6. Varying ETFB/ETFA ratios in plasma/serum of NMD patients and control groups. Boxplots represent the ETFB/ETFA signal ratio in plasma/serum of NMD patients and control groups, where green, yellow, blue and red boxes represent DMDs, BMDs, female carriers and controls, respectively. For each sample group, the box-and-whisker plot represents signal ratio within lower and upper quantile (box), the median (horizontal line within box), percentiles of 5% and 95% (whiskers) and outliers (dots).
