# Supplementary material for: Affinity proteomics within rare diseases: a BIO-NMD study for blood biomarkers of muscular dystrophies
Source: EMBO Mol Med. 2014 Jun 11;6(7):918–36. doi: 10.15252/emmm.201303724 (PMC4119355; doi:10.15252/emmm.201303724)
Supplement: Supplementary file 7 — Supplementary Figure S7 [file emmm0006-0918-SD7.pdf]

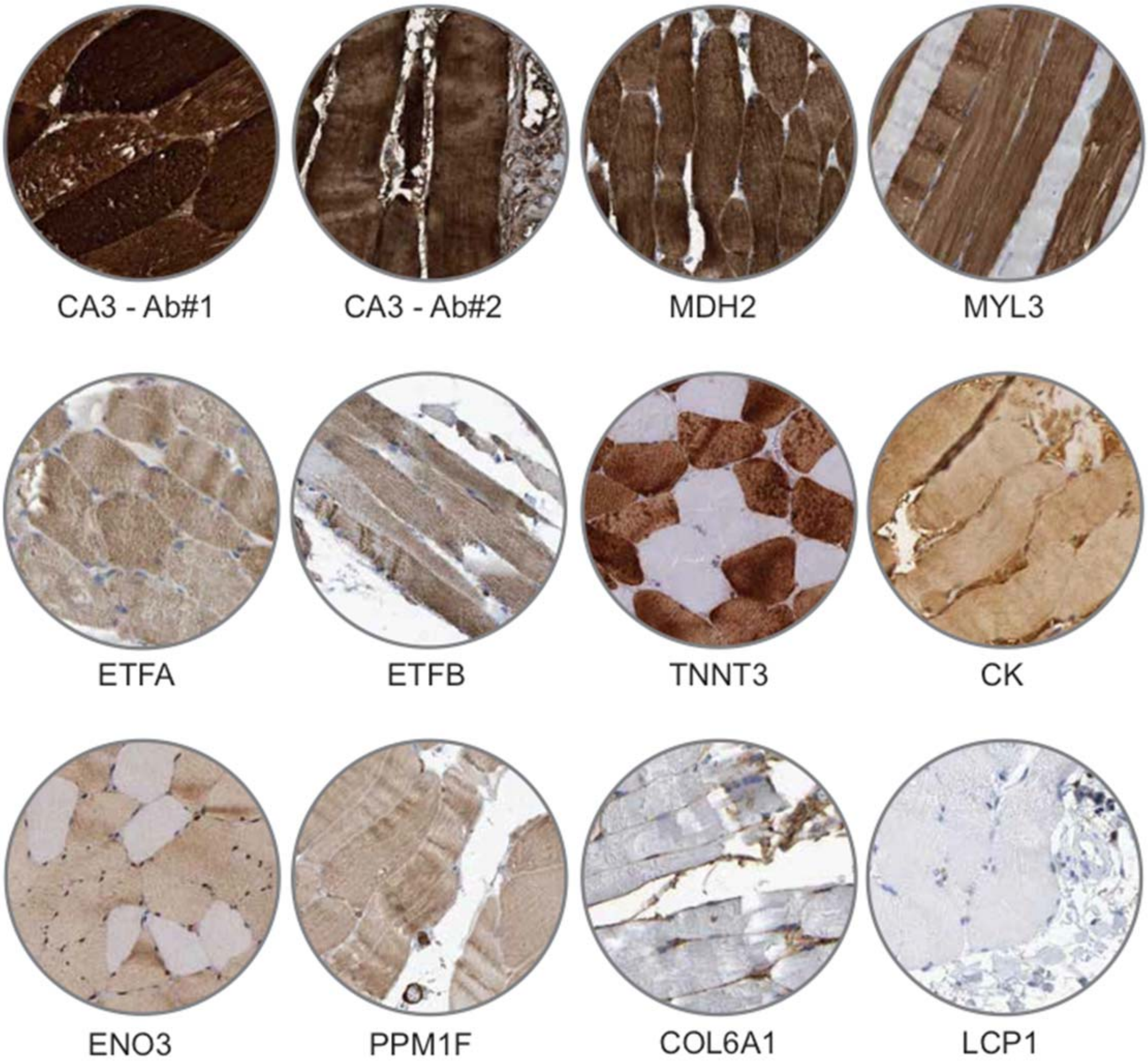

Supplementary Figure S7. Expression of identified protein marker candidates in skeletal muscle tissue. Immunohistochemistry-based protein expression patterns in normal human skeletal muscle for target proteins: CA3, MDH2, MYL3, ETFA, ETFB, TNNT3, CK, ENO3, PPM1F, COL6A1 and LCP1. Note variable expression in different fibre types for MYL3, TNNT3 and ENO3, while CA3, MDH2, ETFA, ETFB, CK and PPM1F appear to be evenly expressed in the cytoplasm of all myocytes.
