# Supplementary material for: Affinity proteomics within rare diseases: a BIO-NMD study for blood biomarkers of muscular dystrophies
Source: EMBO Mol Med. 2014 Jun 11;6(7):918–36. doi: 10.15252/emmm.201303724 (PMC4119355; doi:10.15252/emmm.201303724)
Supplement: Supplementary file 8 — Supplementary Figure S8 [file emmm0006-0918-SD8.pdf]

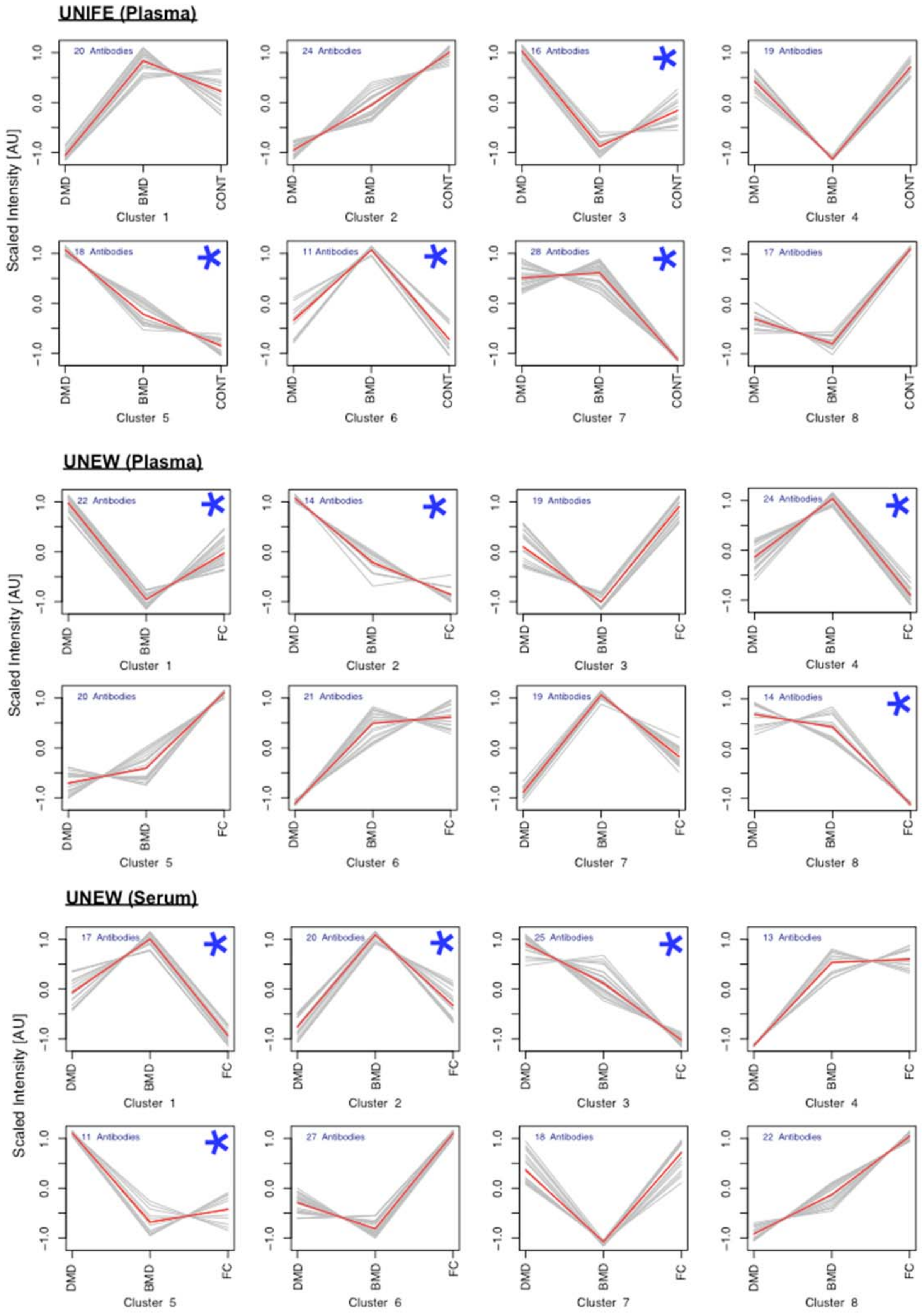

Supplementary Figure S8. Self-organizing tree algorithm (SOTA) clusters summarizing the protein profile trends targets across DMD, BMD patients and controls for 153 antibodies targeting 112 unique muscle-specific proteins. Protein profile trends across DMD, BMD and CONT/FC groups for all of the muscle-specific targets included in the analysis were dissected using SOTA analysis where 8 clusters were chosen to summarize the

different profiles in UNEW plasma and UNEW serum/plasma samples. Each line represents centered and scaled median MFI values across sample groups per antibody and the clusters with a star include antibodies revealing protein profiles with a “DMD increased” and/or “BMD increased” trend as compared to the control groups. In UNIFE cohort there were a total of 65 such proteins (targeted by 73 antibodies) and in UNEW plasma and serum cohorts there were a total of 62 such proteins (targeted by 74 and 73 antibodies, respectively). The union of these 3 sets of “DMD/BMD increased” proteins consisted of 94 (out of 112) targets and the intersection of these 3 sets consisted of 28 targets, including the above highlighted candidates MYL3, CA3, MDH2, ETFA but also other muscle-specific targets such as dystrophin (DMD) or actinin 2 (ACTN2).
