# Supplementary material for: Affinity proteomics within rare diseases: a BIO-NMD study for blood biomarkers of muscular dystrophies
Source: EMBO Mol Med. 2014 Jun 11;6(7):918–36. doi: 10.15252/emmm.201303724 (PMC4119355; doi:10.15252/emmm.201303724)
Supplement: Supplementary file 9 — Supplementary Figure S9 [file emmm0006-0918-SD9.pdf]

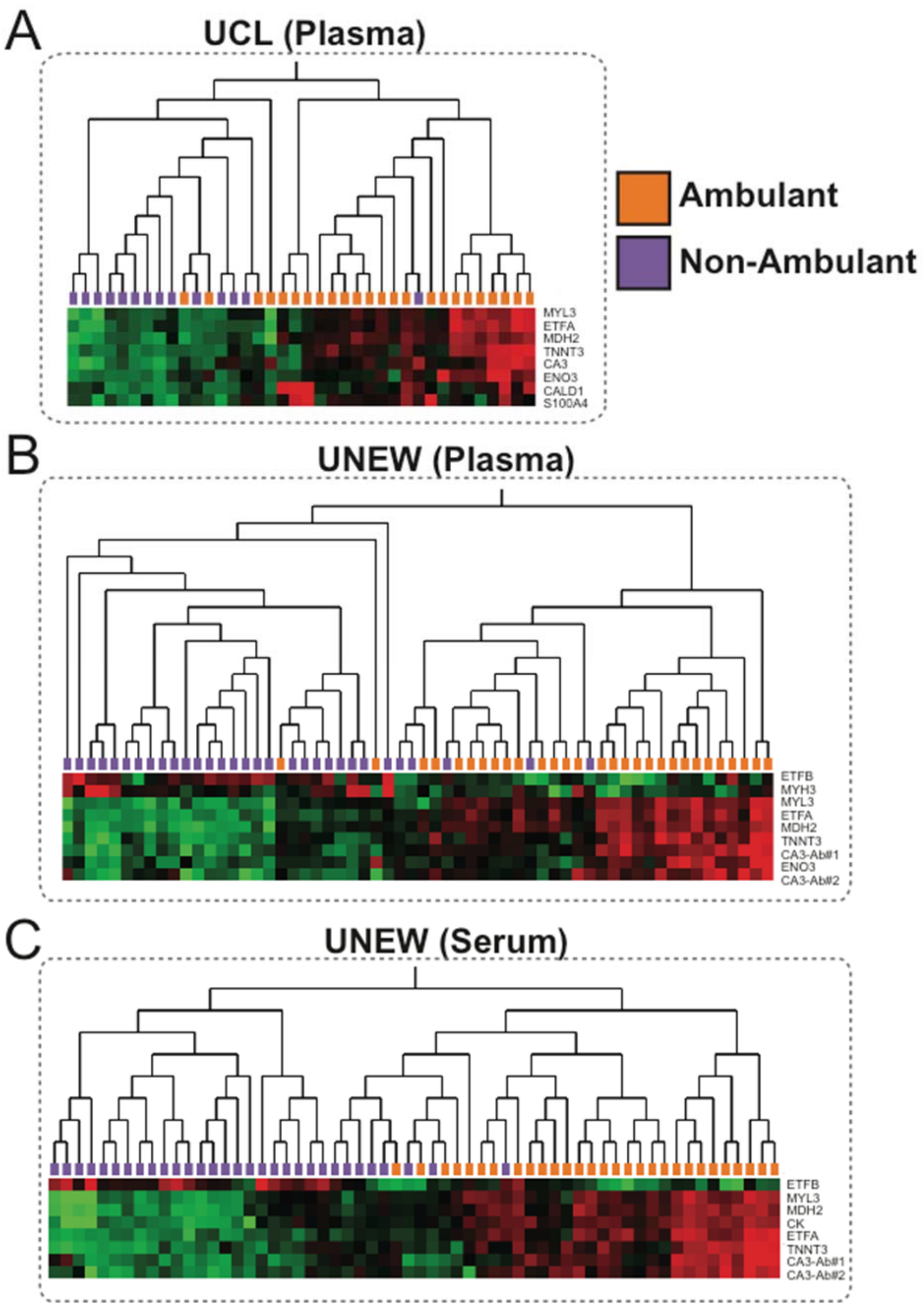

Supplementary Figure S9. Exploratory multi-protein signatures in plasma and/or serum of ambulant and non-ambulant muscular dystrophy patients. Hierarchical clustering of protein profiles representing the main contributors for the grouping of ambulant and non-ambulant muscular dystrophy patient plasma samples collected from UCL **(A)** and UNEW **(B)** and serum from UNEW **(C)**.
