# Supplementary material for: Affinity proteomics within rare diseases: a BIO-NMD study for blood biomarkers of muscular dystrophies
Source: EMBO Mol Med. 2014 Jun 11;6(7):918–36. doi: 10.15252/emmm.201303724 (PMC4119355; doi:10.15252/emmm.201303724)
Supplement: Supplementary file 10 — Supplementary Figure S10 [file emmm0006-0918-SD10.pdf]

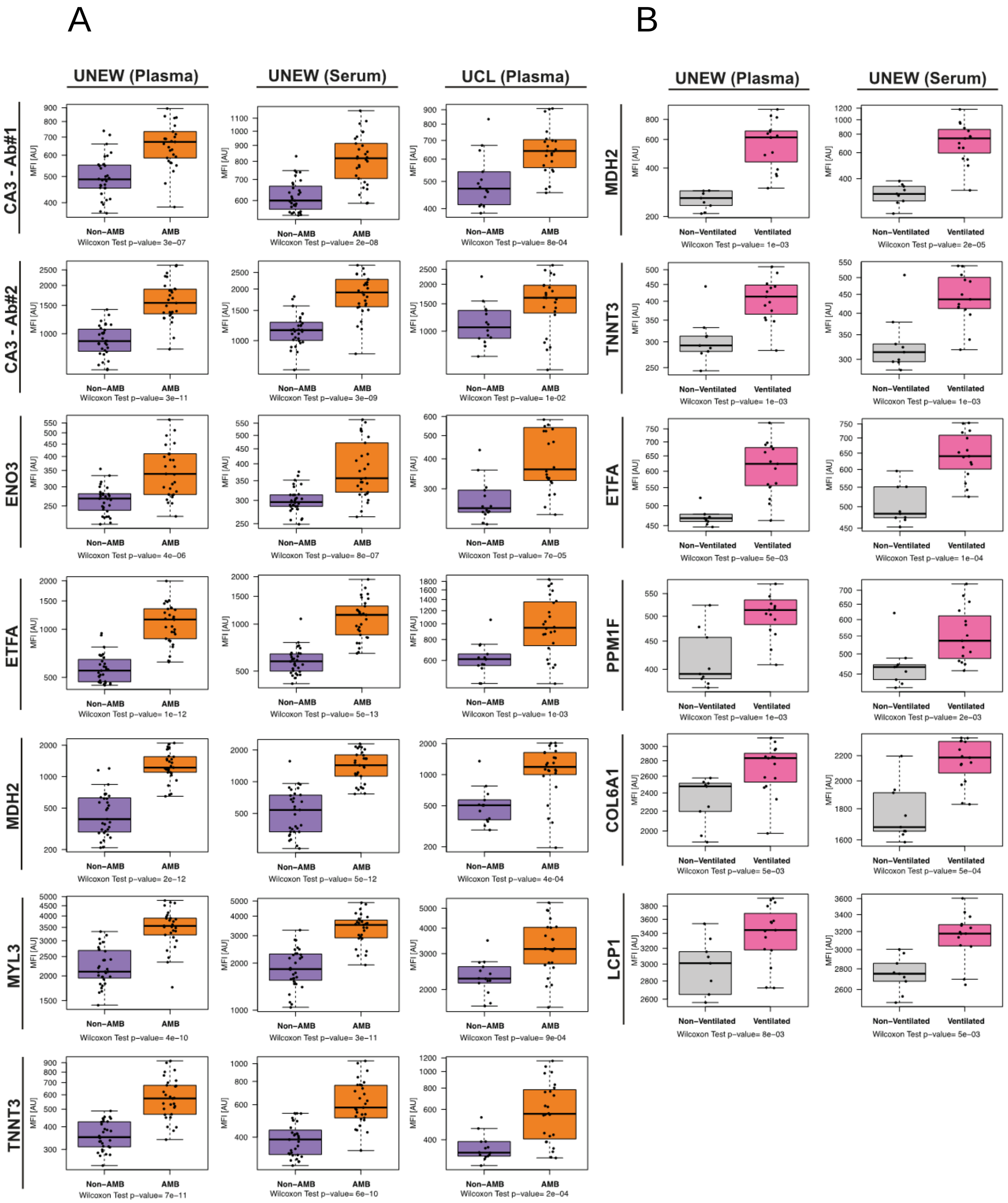

Supplementary Figure S10. Comparison of protein profiles of patients with different ambulatory and respiratory capacity. Boxplots represent the concordant protein profiles significantly differing between ambulant and non-ambulant **(A)** and ventilated and non-ventilated DMD patients **(B)**. MFI values for CA3, ENO3, ETFA, MDH2, MYL3 and

TNNT3 in plasma and/or serum of ambulant and non-ambulant DMD patients are shown, where violet and orange boxes in different cohorts represent non-ambulant and ambulant DMD patients, respectively **(A)**. MFI values for MDH2, TNNT3, ETFA, PPM1F, COL6A1 and LCP1 in plasma and serum of ventilated and non-ventilated DMD patients from UNEW are shown, where grey and pink boxes represent non-ventilated and ventilated DMD patients, respectively **(B)**. For each sample group, the box-and-whisker plot represents signal intensities within lower and upper quantile (box), the median (horizontal line within box), percentiles of 5% and 95% (whiskers) and outliers (dots).
