# Supplementary material for: Affinity proteomics within rare diseases: a BIO-NMD study for blood biomarkers of muscular dystrophies
Source: EMBO Mol Med. 2014 Jun 11;6(7):918–36. doi: 10.15252/emmm.201303724 (PMC4119355; doi:10.15252/emmm.201303724)
Supplement: Supplementary file 11 — Supplementary Figure S11 [file emmm0006-0918-SD11.pdf]

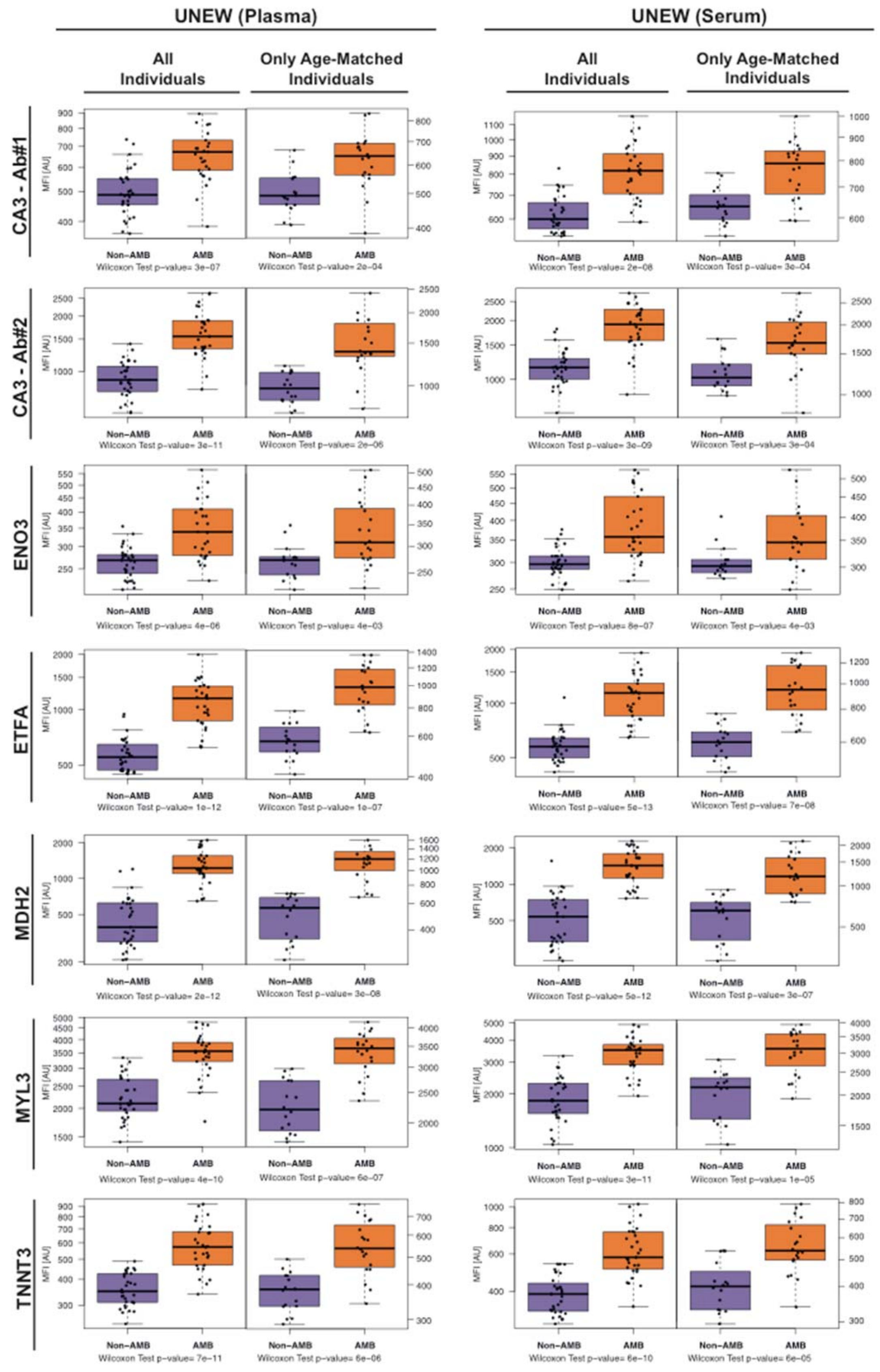

Supplementary Figure S11. Concordance of the comparisons regarding ambulation status based on all or only age-matched patients. Boxplots represent the six concordant protein profiles significantly differing between ambulant and non-ambulant DMD patients independent of age interval of the patients included in the analysis. Violet and orange boxes represent non-ambulant and ambulant UNEW DMD patients, respectively. For each sample

group, the box-and-whisker plot represents signal intensities within lower and upper quantile (box), the median (horizontal line within box), percentiles of 5% and 95% (whiskers) and outliers (dots).
