# Supplementary material for: Affinity proteomics within rare diseases: a BIO-NMD study for blood biomarkers of muscular dystrophies
Source: EMBO Mol Med. 2014 Jun 11;6(7):918–36. doi: 10.15252/emmm.201303724 (PMC4119355; doi:10.15252/emmm.201303724)
Supplement: Supplementary file 13 — Supplementary Table S1 [file emmm0006-0918-SD13.pdf]

Supplementary Table S1. Number and percentage of antibodies revealing signal intensities at noise level. Beads prepared with a protein-free solution and with normal rabbit IgG (without a defined specificity) were used to assess the non-specific background binding. The spread of non-specific binding across all samples was then used to determine the "noise" threshold in serum and plasma (***Supplementary Figure S2***). The number and percentage of antibodies revealing signal intensities below this threshold are summarized in the table. A total of 69 antibodies, namely 18% of the antibodies in the entire set revealed signal intensities at noise level.

	**Nr of Antibodies**		**% of Antibodies**	
	Plasma	Serum	Plasma	Serum
Below threshold for empty bead	16	23	4	6
Below threshold for rabbitIgG bead	66	53	17	14
