# Supplementary material for: Affinity proteomics within rare diseases: a BIO-NMD study for blood biomarkers of muscular dystrophies
Source: EMBO Mol Med. 2014 Jun 11;6(7):918–36. doi: 10.15252/emmm.201303724 (PMC4119355; doi:10.15252/emmm.201303724)
Supplement: Supplementary file 14 — Supplementary Table S2 [file emmm0006-0918-SD14.pdf]

Supplementary Table S2. Correlation between levels of ETFA and ETFB. Spearman's Rho for correlation between the proteins ETFA and ETFB levels in each sample group is shown. Spearman's Rho < -0.5 are highlighted in grey.

Diagnosis	Sample Origin	Sample Type	Correlation btw ETFA and ETFB (Spearman's Rho)
DMD	UNEW	Plasma	**-0.53**
		Serum	**-0.57**
	LUMC	Serum	-0.45
	UCL	Plasma	-0.42
	UNIFE	Plasma	-0.28
BMD	UNEW	Plasma	-0.47
		Serum	-0.25
	UNIFE	Plasma	**-0.70**
FC	UNEW	Plasma	-0.17
		Serum	-0.24
CONT	UNIFE	Plasma	0.05
