# Supplementary material for: Affinity proteomics within rare diseases: a BIO-NMD study for blood biomarkers of muscular dystrophies
Source: EMBO Mol Med. 2014 Jun 11;6(7):918–36. doi: 10.15252/emmm.201303724 (PMC4119355; doi:10.15252/emmm.201303724)
Supplement: Supplementary file 15 — Supplementary Table S3 [file emmm0006-0918-SD15.pdf]

Supplementary Table S3. Correlation of identified protein marker candidates with age. Spearman's Rho for each identified protein marker candidate in each sample group is shown. Spearman's Rho < -0.5 and Spearman's Rho > 0.5 are highlighted in grey.

Diagnosis	Sample Origin	Sample Type	Correlation with Age (Spearman's Rho)							
			CA3 Ab#1	CA3 Ab#2	MDH2	MYL3	ETFA	ETFB	TNNT3	CK
DMD	UNEW	Plasma	**-0.73**	**-0.59**	**-0.82**	**-0.72**	**-0.84**	0.41	**-0.70**	-0.42
		Serum	**-0.69**	**-0.64**	**-0.83**	**-0.84**	**-0.85**	0.49	**-0.69**	**-0.82**
	LUMC	Serum	**-0.73**	**-0.61**	**-0.58**	**-0.75**	**-0.61**	0.33	**-0.53**	**-0.70**
	UCL	Plasma	**-0.61**	**-0.62**	**-0.70**	**-0.74**	**-0.76**	0.18	**-0.63**	**-0.53**
	UNIFE	Plasma	-0.46	-0.39	**-0.77**	**-0.81**	**-0.84**	0.24	**-0.69**	-0.22
BMD	UNEW	Plasma	-0.48	**-0.54**	**-0.59**	**-0.57**	**-0.71**	0.30	**-0.57**	**-0.60**
		Serum	-0.31	-0.43	**-0.56**	**-0.57**	**-0.59**	0.23	**-0.59**	**-0.63**
	UNIFE	Plasma	0.05	-0.17	**-0.67**	**-0.72**	**-0.90**	**0.72**	-0.30	-0.20
FC	UNEW	Plasma	-0.02	-0.16	**-0.61**	**-0.52**	-0.24	0.36	-0.36	0.19
		Serum	0.07	0.00	**-0.57**	**-0.53**	-0.47	0.37	-0.28	**-0.68**
CONT	UNIFE	Plasma	0.20	-0.11	0.28	0.05	0.13	0.06	0.12	-0.15
