# Supplementary material for: Affinity proteomics within rare diseases: a BIO-NMD study for blood biomarkers of muscular dystrophies
Source: EMBO Mol Med. 2014 Jun 11;6(7):918–36. doi: 10.15252/emmm.201303724 (PMC4119355; doi:10.15252/emmm.201303724)
Supplement: Supplementary file 16 — Supplementary Table S4 [file emmm0006-0918-SD16.pdf]

Supplementary Table S4. Number of unique protein targets and number of antibodies per target. Excluding the assay control analytes, the antibody set used in this study consisted of 380 antibodies targeting 315 unique proteins. For 56 out of 315 targets, there were two or more antibodies available in the set, which were raised towards different parts of their respective protein targets.

	Nr of Unique Protein Targets	Nr of Antibodies per Target	Nr of Antibody Pairs per Target	Nr of Antibodies
	259	1		259
	48	2	48	96
	7	3	21	21
	1	4	6	4
**TOTAL:**	**315**		**75**	**380**
