# Supplementary material for: Affinity proteomics within rare diseases: a BIO-NMD study for blood biomarkers of muscular dystrophies
Source: EMBO Mol Med. 2014 Jun 11;6(7):918–36. doi: 10.15252/emmm.201303724 (PMC4119355; doi:10.15252/emmm.201303724)
Supplement: Supplementary file 12 — Supplementary Figure S12 [file emmm0006-0918-SD12.pdf]

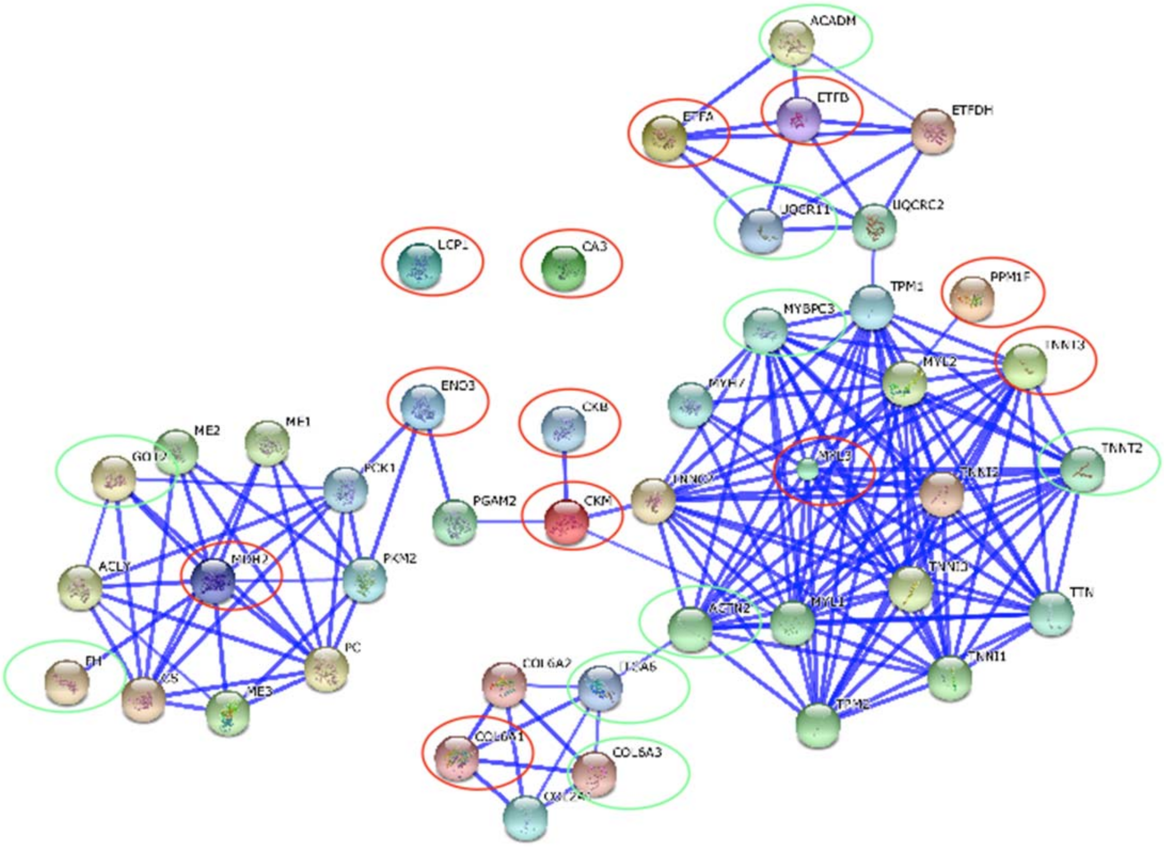

Supplementary Figure S12. STRING analysis of the eleven proteins found to be associated with disease severity in muscular dystrophy. The eleven protein targets listed in *Table 2* were used as STRING input to obtain an interaction network with up to 25 interactors for these 11 targets with a confidence score of 0.8. Although not directly and closely interacting with each, these eleven targets (circled in red) and other targets that were included in the study but revealed statistically less significant differential profiles (circled in green) are not entirely unrelated. Reasons for why only distinct candidates have been found and all included proteins interacting with each other can include (i) lack of statistical power in the analysis due to small sample sizes (ii) the analytical accessibility of these other proteins post leakage or secretion, (iii) the performance of the used antibodies to target these, as well as (iv) the sensitivity of the method.
